# Conditional Survival Analysis of Patients With Locally Advanced Laryngeal Cancer: Construction of a Dynamic Risk Model and Clinical Nomogram

**DOI:** 10.1038/srep43928

**Published:** 2017-03-09

**Authors:** Multidisciplinary Larynx Cancer Working Group, Tommy Sheu, David M. Vock, Abdallah S. R. Mohamed, Neil Gross, Collin Mulcahy, Mark Zafereo, G. Brandon Gunn, Adam S. Garden, Parag Sevak, Jack Phan, Jan S. Lewin, Steven J. Frank, Beth M. Beadle, William H. Morrison, Stephen Y. Lai, Katherine Hutcheson, G. Elisabeta Marai, Guadalupe M. Canahuate, Merrill Kies, Adel El-Naggar, Randal S. Weber, David I. Rosenthal, Clifton D. Fuller

**Affiliations:** 1 Department of Radiation Oncology, The University of Texas MD Anderson Cancer Center, Houston, Texas, USA; 2 Division of Biostatistics, University of Minnesota School of Public Health, Minneapolis, Minnesota, USA; 3 Department of Clinical Oncology and Nuclear Medicine, University of Alexandria, Alexandria, Egypt; 4 Department of Head and Neck Surgery, The University of Texas MD Anderson Cancer Center, Houston, Texas, USA; 5 Department of Radiation Oncology, Henry Ford Hospital, Detroit, MI, USA; 6 Department of Computer Science, University of Illinois at Chicago, Chicago, Illinois, USA; 7 Department of Electrical & Computer Engineering, University of Iowa, Iowa City, IA, USA; 8 Department of Medical Oncology, The University of Texas MD Anderson Cancer Center, Houston, Texas, USA; 9 Department of Pathology, The University of Texas MD Anderson Cancer Center, Houston, Texas, USA

## Abstract

Conditional survival (CS), the survival beyond a pre-defined time interval, can identify periods of higher mortality risk for patients with locally advanced laryngeal cancer who face treatment-related toxicity and comorbidities related to alcohol and smoking in the survivorship setting. Using Weibull regression modeling, we analyzed retrospectively abstracted data from 638 records of patients who received radiation to identify prognostic factors for overall survival (OS) and recurrence free survival (RFS) for the first 3 years of survival and for OS conditional upon 3 years of survival. The CS was iteratively calculated, stratifying on variables that were statistically significant on multivariate regression. Predictive nomograms were generated. The median total follow up time was 175 months. The 3- and 6- year actuarial overall survival (OS) was 68% (95% confidence interval [CI] 65–72%) and 49% (CI 45–53%). The 3-year conditional overall survival (COS) at 3 years was 72% (CI 65–74%). Black patients had worse COS over time. Nodal disease was significantly associated with recurrence, but after 3 years, the 3-year conditional RFS converged for all nodal groups. In conclusion, the CS analysis in this patient cohort identified subgroups and time intervals that may represent opportunities for intervention.

The American Cancer Society estimates that there will be 13,560 new cases of and 3,640 deaths attributable to laryngeal cancer in the United States annually^1^. Since the seminal 1991 VA larynx study showed overall survival equivalence between primary surgical and non-surgical treatment, there has been a shift toward organ preservation, particularly in moderate to very locally advanced tumors; however, nationally there has also been an increase in overall mortality^2^,^3^. Airway compromise, infection, failure to thrive, or complications from invasive procedures are major contributors to early mortality. With upfront surgical resection, the hospital readmission rate within 30 days can be as high as 27%; although, at high volume centers, mortality during admission for surgery is low and approaches 1%^4^,^5^. Medical comorbidities including smoking history, alcohol use, second primaries, or long-term treatment-related complications such as chronic, silent aspiration or carotid artery atherosclerosis-related stroke emerge as significant causes of mortality later in the disease course^6^.

Larynx cancer patients may have a reduction in the malignancy-related hazard of death with longer survival, but face increasing hazards from unrecognized late toxicities or cumulative/synergistic sequelae of continued tobacco or alcohol use. It is standard practice to report survival probabilities from the time of diagnosis. Analyses incorporating the endpoint of conditional survival (CS), that is survival that is conditional on surviving a certain amount of time from diagnosis, can provide time-specific prognostic information as well as a nuanced and time-dependent assessment of the mortality risks associated with increased survivorship. CS analysis has been implemented for various intervals for several other cancer sites including: brain, gastrointestinal, lung, breast, prostate, and the ovaries, but there have been no reports of CS for patients with locally advanced laryngeal cancer^7^,^8^,^9^,^10^,^11^.

The aims of this study are as follows: 1) to generate a robust, parametric survival prediction model formulated with patient characteristics available at the time of diagnosis; 2) to apply this model to predict conditional overall survival (COS) and to assess the influence of prognostic factors in the CS setting; and 3) to generate clinically usable nomograms that can used for real-time individual risk estimation at the point of diagnosis and at follow up.

## Methods and Materials

### Data Selection

This study was approved by the institutional review board of the University of Texas MD Anderson Cancer Center. A waiver of consent was granted for retrospective data analysis such as in the current study. Data from charts of patients who were treated with adjuvant or definitive radiotherapy for locally advanced (AJCC version 7, T3: larynx confined tumor with vocal cord immobilization or invasion into post-cricoid, paraglottic, pre-epiglottic, or inner thyroid cartilage areas, and T4: local invasion into thyroid cartilage or beyond the larynx including the carotid artery or pre-vertebral space) laryngeal cancer at The University of Texas MD Anderson Cancer Center between 1983 and 2011 were retrospectively extracted; the details of this data set are reported elsewhere^12^,^13^,^14^. Overall and recurrence free survival data as well as potential prognostic covariates such as age, sex, ethnicity, Eastern Cooperative Oncology Group (ECOG) performance status, primary site, T classification, and treatment details were incorporated into our analysis based on reports of these being significant prognosticators in the previously published literature^15^,^16^,^17^,^18^,^19^.

### Overall Survival Analysis

All statistical analysis was performed using Stata (College Station, TX). For the OS regressions, *t*_*0*_ was set at the time of diagnosis and all patients were censored at 3 years. For the conditional overall survival (COS) regressions, given that a patient survives *t* years (*t* = 3 years for this study), the COS was defined as the probability that the patient will survive an additional *Δt* years (*Δt* = 3 years for this study)^11^. The 3 year time intervals were selected in order to balance the total amount of time-at-risk between the OS and COS analyses. In an effort to minimize total time at-risk difference between the conventional and COS analyses, if an event was not recorded by the 6-year follow up point, the patient was censored for regression analyses. For recurrence free survival (RFS), a failure was defined as either death or local/distant recurrence.

Multivariable Weibull regression models were fitted for the 3 year OS, 3 year COS, and RFS endpoints. Because the Weibull model is an accelerated failure time model, estimates of survival probabilities are frequently more robust to departures from the proportional hazards assumption, which relaxed the restrictions on covariate selection. Age, sex, race (White, Black or other), site (glottic vs. supraglottic, transglottic or subglottic), T category, N category, smoking status, ECOG performance status, local therapy (radiotherapy alone, laryngectomy with postoperative radiotherapy, or chemoradiation), and chemotherapy (no chemotherapy, induction chemotherapy, concurrent chemotherapy or induction followed by concurrent) were included based on prior analysis^13^. Restricted cubic splines were used to model nonlinear relationships between the covariates and the log hazard (equivalently log acceleration factor) as needed. The 3-year COS and 3-year conditional RFS (CRFS) at 6 month intervals for the first 3 years of follow up was calculated, stratifying by covariates that were significant on the multivariate regression.

### Prediction Models and Nomograms

In an effort to reduce over-fitting for the survival prediction models, an Akaike Information Criteria (AIC) reduction algorithm was used to select the covariates that would generate the most parsimonious model for survival prediction^20^. The selected covariates were incorporated into a Cox proportional hazards prediction model that was used to formulate predictive nomograms for overall survival (OS) and overall survival conditional upon 3 years of survival (COS). The proportional hazards assumption for the prediction models was verified using Schoenfeld residuals with the null hypothesis of a slope of zero when scaled Schoenfeld residuals were regressed over time. Failure to reject this hypothesis was considered verification of the proportional hazards assumption. The fit was assessed using Cox-Snell residuals plotted against the Nelson-Aalen cumulative hazard estimate. From these models, nomographic representations of the 1 and 3 year overall survival, and 1 and 3 year overall survival conditional upon 3 years of survival (4 and 6 years of total survival respectively) were generated^21^.

## Results

### Patient and Disease Characteristics

An existing database of 638 patients treated with radiotherapy for locally advanced (T3 or T4 disease) laryngeal cancer between 1983 and 2011 was used. Further information regarding patient characteristics, treatment specifics, and survival analyses for this database have been reported previously^12^,^13^,^14^. Ten records were excluded for being incomplete, and 13 patients treated with induction chemotherapy, surgery and postoperative radiotherapy were also excluded in an effort to reduce study population heterogeneity and because the total number of patients meeting these criteria was small. A total of 615 usable patients’ datasets remained (Table 1).

The mean age was 59 years and the study population was 74% male. The majority of patients presented with disease in the supraglottic sub-site (67%) with the glottic sub-site (17%) being the next most common. More patients (66%) presented with T3 disease, and fifty-six percent had evidence of lymph node involvement. Patients with a history of smoking comprised 94% of the study population. The patient and disease characteristics appeared similar between all patients presenting at the time of diagnosis and the 3 years survivors comprising the CS cohort.

### Treatment Details

Most patients (44%, N = 270) were treated initially with surgery followed by postoperative radiation (N = 241) or chemoradiation (N = 29). Thirty percent of T3 patients were treated with definitive surgery compared to 73% of T4 patients. Induction chemotherapy was given to 63 of the 192 patients and 40 of the 153 patients treated with radiation and chemoradiation, respectively. Thirty-six of 191 (19%) patients treated with radiation (with or without induction chemotherapy) and 24 out of 153 (16%) patients treated with chemoradiation (with or without induction chemotherapy) went on to receive salvage surgery. Eleven percent of patients with T3 disease went on to receive salvage surgery compared to 8% patients with T4 disease. More information regarding treatment details can be found in Table 1.

### Survival Results

**Figure 1:**
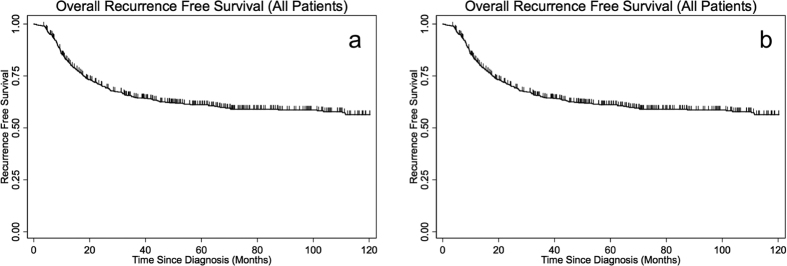
(**a**) Overall survival of all patients. (**b**) Recurrence free survival of all patients.

The total median follow-up time was 175 months, and at the time of data collection 28% of the study population was still alive. The median overall survival time for all patients was 71.0 months (95% confidence interval [CI] 61.1–81.2 months). The OS and RFS for all patients are depicted in Fig. 1. The median overall survival was 67.7 months (CI 51.1–91.1 months), 62.2 months (CI 50.3–78.6 months), and 80.8 months (CI 70.2–101.9 months) for patients treated with radiation alone, surgery followed by postoperative (chemo)radiation, and definitive chemoradiation alone respectively. At 10 years, 40% of patients had had local or distant progression of disease with a median time to recurrence of 13.3 months (CI 11.4–15.5 months). There was no difference in RFS between the patients treated sequentially with induction chemotherapy followed by concurrent chemoradiation compared to those treated with concurrent chemoradiation alone (log rank p-value = 0.622).

### Conditional Survival Results

**Figure 2:**
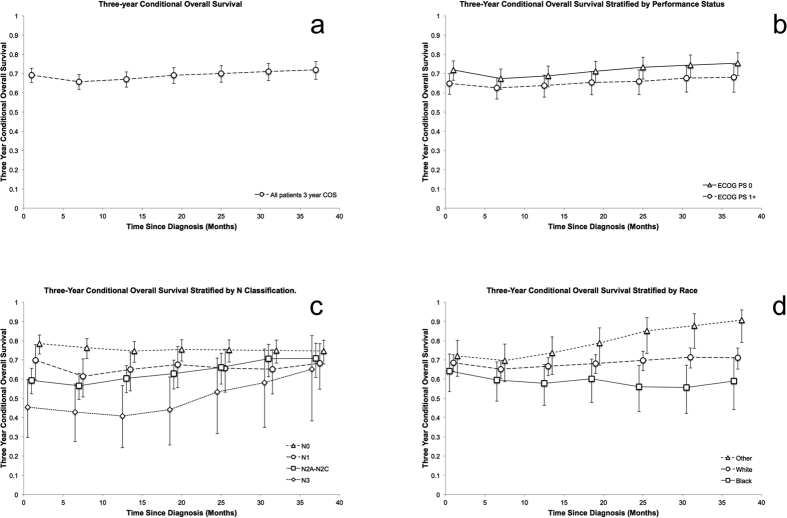
(**a**) Three-year conditional overall survival calculated at 6 month intervals. (**b**) Three-year conditional overall survival stratified by performance status calculated at 6 month intervals. (**c**) Three-year conditional overall survival stratified by N classification calculated at 6 month intervals. (**d**) Three-year conditional overall survival stratified by race calculated at 6 month intervals.

**Figure 3:**
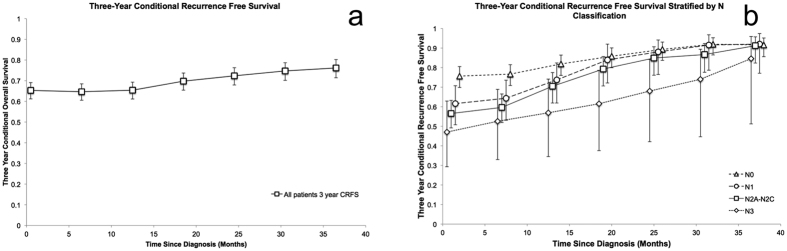
(**a**) Three-year conditional recurrence free survival for all patients calculated at 6 month intervals. (**b**) Three year conditional recurrence free survival stratified by N classification calculated at 6 month intervals.

The 3- and 6-year actuarial survivals from the time of diagnosis for all patients were 68% (CI 65–72%) and 49% (CI 45–53%) respectively. Among 3 year survivors, the 3-year COS (6 years of survival total) was 72 percent (CI 65–74%), a 23% increase compared to the 6 year overall survival of all patients (Fig. 2a). COS stratified by prognosticators significant on multivariate analysis were calculated and select prognosticators are shown Fig. 2. There was no appreciable difference in trend or separation based on performance status (Fig. 2b). When stratified by nodal status, there was a trend toward improved 3-year COS in more advanced nodal disease with relative stability of patient’s with N0 and N1 disease (Fig. 2c). The three-year COS worsened with increase follow up for Black patients, remained constant for White patients, and improved for all other patients (Fig. 2d). The 6 month incremental CRFS for all patients is shown in Fig. 3a. Figure 3b shows the 6 month incremental CRFS stratified by nodal status, which demonstrates a pattern of convergence similar to the COS stratified by nodal status.

### Multivariate Modeling

**Figure 4:**
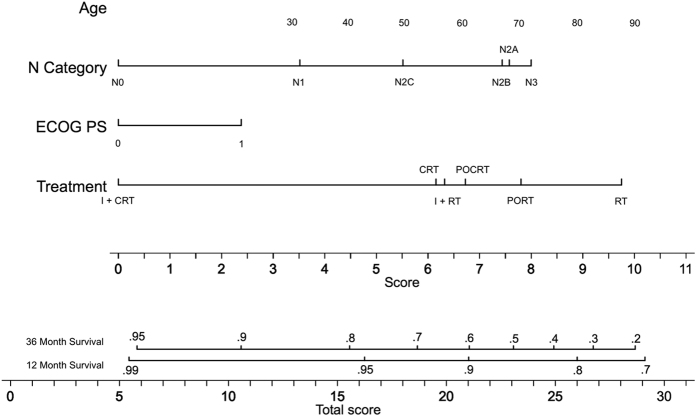
Nomogram for calculating a 1 year and 3 year overall survival prediction from the time of diagnosis. For each parameter, a vertical line is drawn intersecting with the value corresponding to the patient and the “Score” line. In order to determine the 12 or 36 month prediction, a vertical line is drawn intersecting the cumulative sum of all the “Score” line intersection values on the “Total Score” line with the corresponding survival line. Abbreviations: I – induction chemotherapy; CRT – chemoradiation; POCRT – postoperative chemoradiation; PORT – postoperative radiation; RT – radiation.

**Figure 5:**
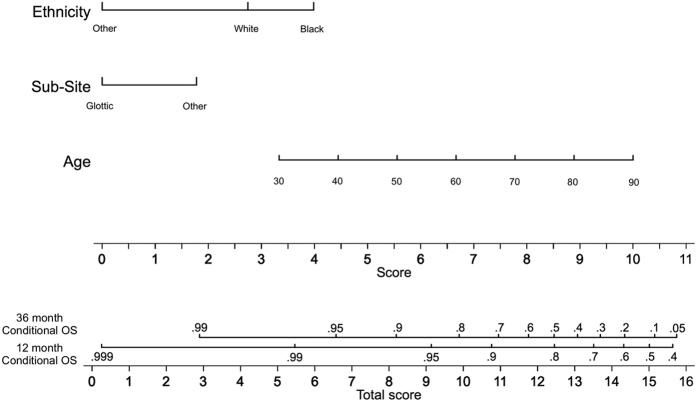
Nomogram for calculating an overall 4 year and 6 year survival prediction conditional upon 3 years of survival. For each parameter, a vertical line is drawn intersecting with the value corresponding to the patient and the “Score” line. In order to determine the 12 or 36 month prediction, a vertical line is drawn intersecting the cumulative sum of all the “Score” line intersection values on the “Total Score” line with the corresponding survival line. Abbreviations: I – induction chemotherapy; CRT – chemoradiation; POCRT – postoperative chemoradiation; PORT – postoperative radiation; RT – radiation.

Three multivariate regressions using a Weibull distribution model were performed with the results detailed in Tables 2, 3 and 4. For these regressions, calibration plots demonstrating the relationship between the Nelson Aalen cumulative hazard estimate and the Cox-Snell residuals are shown in Supplemental Figs S1–S3. For the adjusted initial 3-year OS regression (Table 2, all patients censored at 3 years, total at risk time of 18,561 months), significant prognosticators were age (p = 0.031), N category (p < 0.001), performance status (p = 0.019), treatment with induction followed by chemoradiation therapy (p = 0.001) and treatment with chemoradiation (p = 0.041). The cumulative time at risk for this cohort was 18,561 years. For the adjusted 3-year COS regression, (Table 3, N = 401, all patients censored at 6 years, total at risk time of 25,650 months), age (p < 0.001), sub-site (p = 0.019), and ethnicity (Black race p = 0.0028, other race p = 0.0009) were statistically significant prognosticators. For the adjusted RFS regression (Table 4), nodal stage (HR 1.21, CI 1.12–1.30, p < 0.001), performance status (HR 1.34, CI 1.01–1.76, p = 0.040), treatment with surgery and postoperative radiation therapy (HR 0.60, CI 0.40–0.89, p = 0.011) and treatment with induction followed by chemoradiation therapy (HR 0.46, CI 0.24–0.90, p = 0.024) were statistically significant. The cumulative time at risk for this cohort was 25,650 years. The results of the Cox proportional hazards regressions represented by the 2 nomograms given in Figs 4 and 5 are shown in Table 5. The proportional hazards assumption was verified; the null hypothesis of slope of zero for a generalized linear regression of the scaled Schoenfeld residuals over time was not rejected (p = 0.95 for the OS regression and p = 0.33 for the COS regression). The calibration plots for these two regressions are shown in Supplemental Figs S4 and S5.

## Discussion

Conditional survival analyses provide more time-dependent prognostic information that better reflects the expected natural history of disease. Based on one of the largest, long-term study cohorts of patients with locally advanced laryngeal cancer, we have generated nomograms that can be used to predict individualized overall survival from diagnosis and conditional overall survival to multiple time points. We have found a statistically significant difference between the 3-year conditional overall survival to 6 years and the 6-year overall survival endpoint. However, when assessed at 6 month intervals over the span of 3 years, there was little change in the COS of the entire cohort of patients, a departure from the typical findings of other conditional survival analyses where patients overall tend to have improved COS with increased follow up. Finally we have found mortality patterns among patient sub-groups that may warrant added scrutiny and intervention.

Age, sub-site, nodal disease, and race are known survival prognosticators for locally advanced laryngeal cancers; however, the time-specific influence of these factors has not been described^16^,^19^,^22^. Black patients were found to have diverging 3-year COS with increased follow up compared to patients of other races. Taken with the observation that ethnicity was not a significant prognosticator of recurrence on adjusted analysis, the observed separation in COS may indicate that the unfavorable COS profile was unrelated to progressive disease. In the multivariate analysis, we found age, nodal burden, performance status, and treatment with chemoradiation (with or without induction) to be significant for 3-year OS. However, in the adjusted 3-year COS analysis, race and larynx sub-site emerged as significant COS prognosticators; whereas, nodal disease burden, performance status and treatment were no longer statistically significant prognosticators. The emergence of race and larynx subsite later in the survivorship may reflect a gap in detection of recurrence or disparity in the management of comorbidities based on healthcare utilization.

Although protracted follow up intervals typically begin at 2 years, patients with advanced (N3) nodal disease who appear to have elevated recurrence risk at least until the third year of follow up may need closer surveillance, as patients with N3 disease only achieved near equivalent CRFS to patients with less nodal burden at the 3-year time mark. Although this trend may point to a reduction in primary laryngeal cancer relate death, generally, in head and neck cancer a different mortality pattern emerges later in the disease course with upwards of 29% of patients developing a second primary cancer between 2–5 years after initial diagnosis^23^. For example in RTOG 90-03, a second primary malignancy was the more likely cause of death among 6-year survivors, and after 6 years the majority of deaths were unrelated to the primary malignancy or treatment-related toxicities^6^. These observations may explain why as a whole, there was not a definite trend toward improved COS during the first 3 years of follow up among our patients.

Patients treated with definitive chemoradiation therapy with or without induction chemotherapy had favorable survival outcomes during the first 3 years, and induction chemotherapy followed by chemoradiation was a significant predictor of improved RFS on adjusted analysis. The adjusted COS analysis showed induction chemotherapy with definitive radiation to be a near significant predictor of survival, whereas chemoradiation treatment was not. The results of the 2013 update of RTOG 91-11 revealed a relative, but non-statistical, increase in non-laryngeal cancer-related death with concurrent chemoradiation compared to induction chemotherapy, perhaps because the initial benefit of laryngeal preservation and or reduction in recurrence conferred by concurrent chemoradiation may be partially offset by increased late mortality arising from treatment-related toxicity^24^. In the context of our dataset, this hypothesis would be better supported if chemoradiation conferred a worse prognosis on COS analysis.

Limitations to this study are typical of retrospective analyses performed at single institutions. The database used to generate these models consisted of patient data from a single tertiary cancer center, and may reflect a patient sample that is not generalizable to the general population. As such the presented nomograms are not yet suitable for general use prior to validation of the predictive models with external datasets. The current study also included a sizable cohort of patients who were treated before the 1991 Department of Veteran Affairs Laryngeal Cancer Study Group, which prompted a shift toward an organ preservation paradigm^3^. Our results are comparable to other reports of similarly treated patients with locally advanced laryngeal cancer, some of which did not detect a survival difference between patients with T3 and T4 category tumors^22^,^25^. In addition we did not correct for multiple statistical testing on the same data set. Although the larger overall time-at-risk may be a plausible explanation for the emergence of new significant prognosticators on the COS analyses, the magnitude of the respective hazard ratios for race and sub-site for years 3–6 compared to the first 3 years suggest that this finding is not solely due to a difference in statistical power. Finally, although the Weibull distribution appeared to fit the 3-year OS and the 3-year COS endpoint regressions well, there appeared to be room for improvement in model selection for the PFS endpoint.

Ideally, evolving risk models will account for a host of time-varying features or dynamic treatment/surveillance decision options in a quantitative manner, rather than an ad hoc approach. To this end, the use of advanced statistical learning, or feature-clustering methods may provide avenues for more granular assessment of individual patient prognostic or predictive evaluation tools for precision medicine approaches to care. These methods can be used to generate highly desirable relevant prognostic information that is pertinent to patients at subsequent follow up visits. The presented nomogram is an example of an easily implementable, institution-specific communication tool that can be used for patient discussion and evidence-based surveillance and risk assessment reflective of local patient demographics and practice patterns.

## Additional Information

**How to cite this article:** Sheu, T. *et al*. Conditional Survival Analysis of Patients With Locally Advanced Laryngeal Cancer: Construction of a Dynamic Risk Model and Clinical Nomogram. *Sci. Rep.* **7**, 43928; doi: 10.1038/srep43928 (2017).

**Publisher's note:** Springer Nature remains neutral with regard to jurisdictional claims in published maps and institutional affiliations.

## Supplementary Material

Supplementary Information

## Figures and Tables

**Table 1:** Patient Characteristics.

	All Patients	3-Year Survivors
	Number (Percentage)	
Median Age	59	58
Male Sex	456 (74)	290 (72)
Subsite		
Glottic	102 (17)	71 (18)
Non-Glottic	513 (83)	330 (82)
T Category		
T3	403 (66)	276 (68)
T4	212 (34)	128 (32)
N Category		
N0	269 (44)	203 (50)
N1	97 (16)	63 (16)
N2A	22 (4)	10 (2)
N2B	89 (14)	51 (13)
N2C	98 (16)	59 (15)
N3	40 (6)	18 (4)
ECOG PS		
0	306 (50)	214 (53)
1–3	309 (50)	190 (47)
Ethnicity		
White	429 (70)	290 (72)
Black	90 (14)	55 (14)
Other	96 (16)	59 (14)
Treatment		
RT	129 (21)	79 (20)
Induction + RT	63 (10)	45 (11)
PORT	241 (39)	152 (38)
POCRT	29 (5)	18 (4)
Induction + CRT	40 (6)	29 (7)
CRT	113 (18)	78 (19)
Chemotherapy		
*Induction*		
Platinum based	103	57
Platinum + Biologic	7	11
Unknown	4	1
*Concurrent*		
Platinum based	157	101
Biologic Alone	13	8
Platinum + Biologic	3	4
Unknown/Other	10	8

**Table 2:** Results of Weibull Parametric Regression For Overall Survival: Years 1–3.

Covariate	95% Confidence		
	HR	Interval	p-value
Age	1.02	1.00–1.03	0.031
Nodal Disease	1.25	1.15–1.35	<0.001
T Category	1.08	0.76–1.54	0.652
Site	1.30	0.80–2.11	0.296
ECOG PS	1.44	1.06–1.95	0.019
Treatment			
Radiation Alone	1.00		
Induction chemotherapy+RT	0.67	0.38–1.19	0.170
PORT	0.78	0.52–1.17	0.224
POCRT	0.59	0.28–1.26	0.173
Induction + CRT	0.22	0.09–0.53	0.001
CRT	0.60	0.37–0.98	0.041
Ethnicity			
White	1.00		
Black	1.09	0.75–1.59	0.639
Other	1.18	0.82–1.69	0.371

N = 615 patients with 18,561 total months at risk.

ECOG PS – European Cooperative Oncology Group performance status.

RT – radiation therapy; PORT – postoperative radiation therapy; POCRT – postoperative chemoradiation therapy; CRT – chemoradiation therapy.

Site –Non-glottic subsite vs. glottic subsite.

Nodal disease –N1, N2A, N2B, N2C, N3 vs. N0.

**Table 3:** Results of Conditional Weibull Parametric Regression For Overall Survival: Years 3–6.

Covariate	95% Confidence		
	HR	Interval	p-value
Age	1.05	1.03–1.08	<0.001
Nodal Disease	1.03	0.91–1.15	0.670
T Category	0.85	0.52–1.38	0.499
Site	2.27	1.14–4.50	0.019
ECOG	1.21	0.81–1.82	0.357
Treatment			
Radiation Alone	1.00		
Induction chemotherapy+ RT	0.42	0.17–1.02	0.055
PORT	1.06	0.62–1.83	0.822
POCRT	1.32	0.50–3.50	0.574
Induction+ CRT	0.59	0.20–1.72	0.333
CRT	0.80	0.44–1.47	0.469
Ethnicity			
White	1.00		
Black	1.73	1.06–2.83	0.028
Other	0.30	0.12–0.74	0.009

N = 401 patients with 25,650 total months at risk.

ECOG PS – European Cooperative Oncology Group performance status.

RT – radiation therapy; PORT – postoperative radiation therapy; POCRT – postoperative chemoradiation therapy; CRT – chemoradiation therapy.

Site –Non-glottic subsite vs. glottic subsite.

Nodal disease N1-N3 vs. N0.

**Table 4:** Weibull Parametric Regression for Recurrence Free Survival.

Covariate	95% Confidence		
	HR	Interval	p-value
Age	1.00	0.98–1.01	0.624
Nodal Disease	1.19	1.11–1.29	<0.001
T Category	1.30	0.94–1.78	0.112
Site	1.03	0.70–1.50	0.894
ECOG	1.36	1.04–1.80	0.025
Treatment			
Radiation Alone			
Induction chemotherapy+ RT	1.03	0.65–1.65	0.954
PORT	0.64	0.43–0.94	0.014
POCRT	1.03	0.55–1.92	0.848
Induction + CRT	0.47	0.24–0.92	0.035
CRT	0.74	0.48–1.14	0.260
Ethnicity			
White			
Black	1.09	0.75–1.59	0.641
Other	1.22	0.85–1.74	0.287

N = 605 patients with 32,491 total months at risk.

ECOG PS – European Cooperative Oncology Group performance status.

RT – radiation therapy; PORT – postoperative radiation therapy; POCRT – postoperative chemoradiation therapy; CRT – chemoradiation therapy.

Site –Non-glottic subsite vs. glottic subsite.

Nodal disease N1-N3 vs. N0.
