# Supplementary material for: Conditional Survival Analysis of Patients With Locally Advanced Laryngeal Cancer: Construction of a Dynamic Risk Model and Clinical Nomogram
Source: Sci Rep. 2017 Mar 9;7:43928. doi: 10.1038/srep43928 (PMC5343446; doi:10.1038/srep43928)

**Conditional Survival Analysis of Patients With Locally Advanced Laryngeal Cancer: Construction of a Dynamic Risk Model and Clinical Nomogram.**

*Multidisciplinary Larynx Cancer Working Group*

Contributing authors:

Tommy Sheu MD, MPH[1], David Vock, PhD[5], Abdallah S. R. Mohamed, MD, MSc[1,6]*, Neil Gross, MD[2], Collin Mulcahy, BS[1], Mark Zafereo, MD[2], G. Brandon Gunn MD[1], Adam S. Garden MD[1], Parag Sevak[7], MD, Jack Phan MD, PhD[1], Jan S. Lewin, PhD[2], Steven J. Frank MD[1], Beth M. Beadle MD, PhD[1], William H. Morrison MD[1], Stephen Y. Lai, MD, PhD[2], Kate Hutcheson, PhD[2], G. Elisabeta Marai, PhD[8], Guadalupe M. Canahuate, PhD[9], Merrill Kies, MD[3], Adel El-Naggar, MD[4], Randal S. Weber, MD[2], David I. Rosenthal MD[1], Clifton D. Fuller MD, PhD[1]*.

[1]Departments of Radiation Oncology, [2]Head and Neck Surgery, [3]Medical Oncology, and [4]Pathology, The University of Texas MD Anderson Cancer Center, Houston, Texas, USA.

[5]Department of Biostatistics, University of Minnesota School of Public Health, Minneapolis, Minnesota, USA.

[6]Department of Clinical Oncology and Nuclear Medicine, University of Alexandria, Alexandria, Egypt.

[7]Department of Radiation Oncology, Henry Ford Hospital, Detroit, MI, USA.

[8]Department of Computer Science, University of Illinois at Chicago, Chicago, Illinois, USA.

[9]Department of Electrical & Computer Engineering, University of Iowa, Iowa City, IA, USA.

**Supplemental Figure**

Supplemental Figure S1

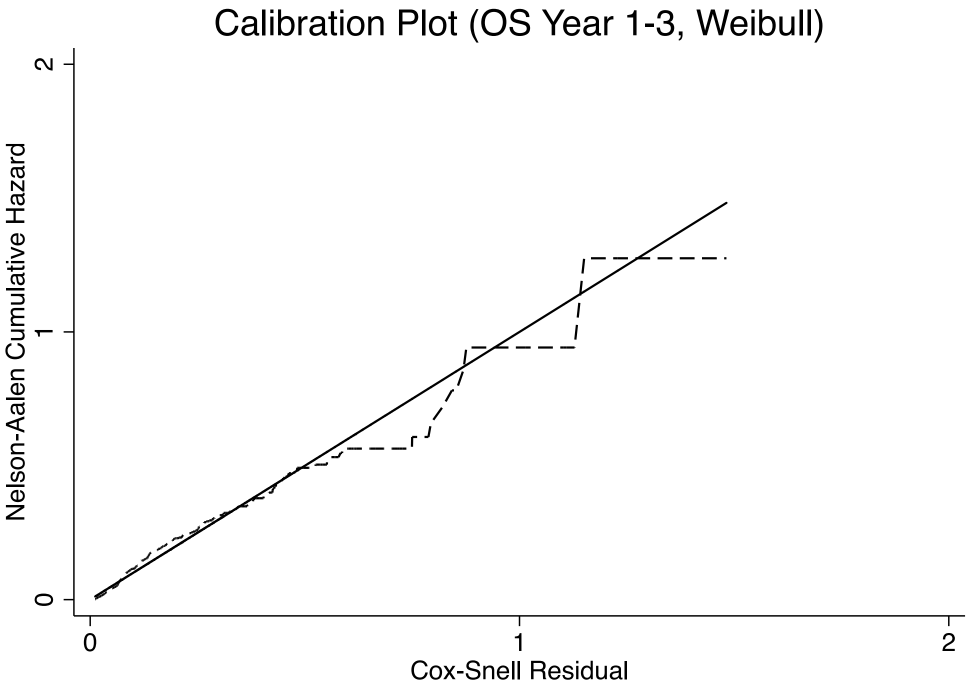


Supplemental Figure S2

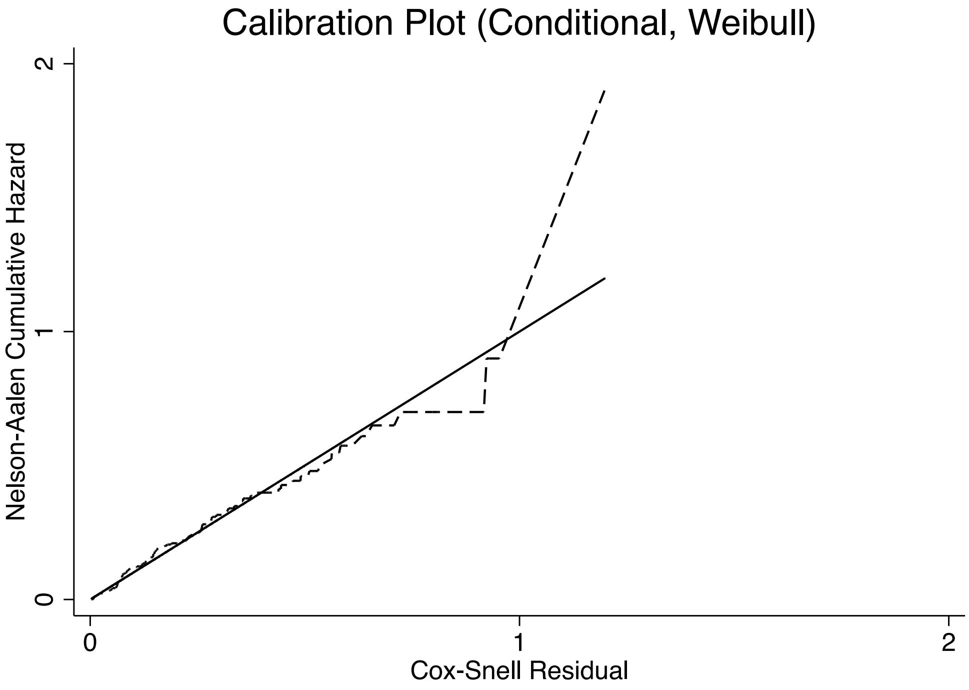

Supplemental Figure S3

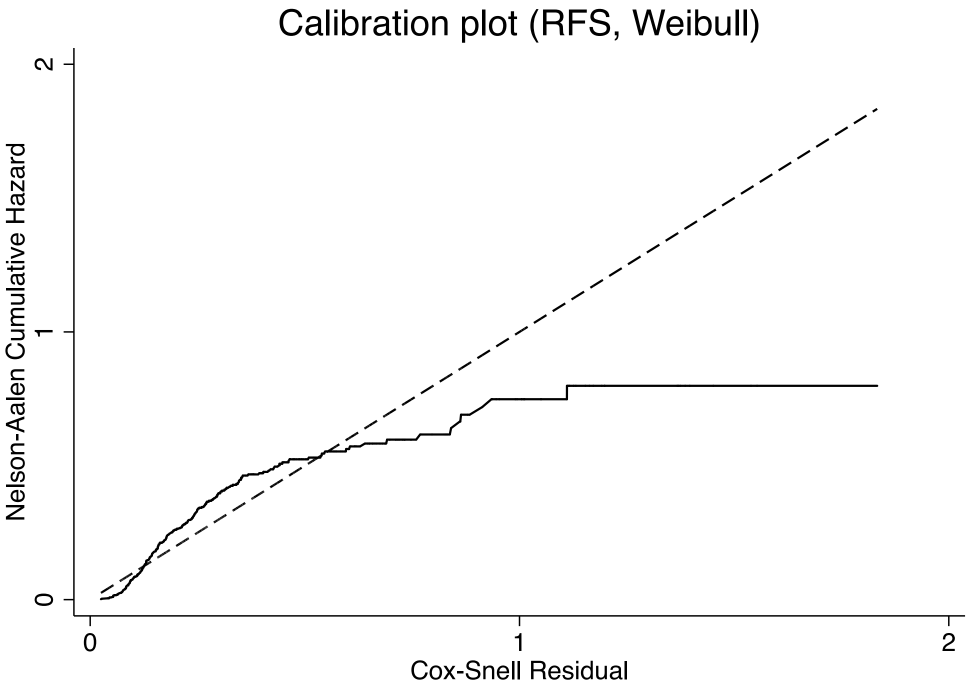


Supplemental Figure S4

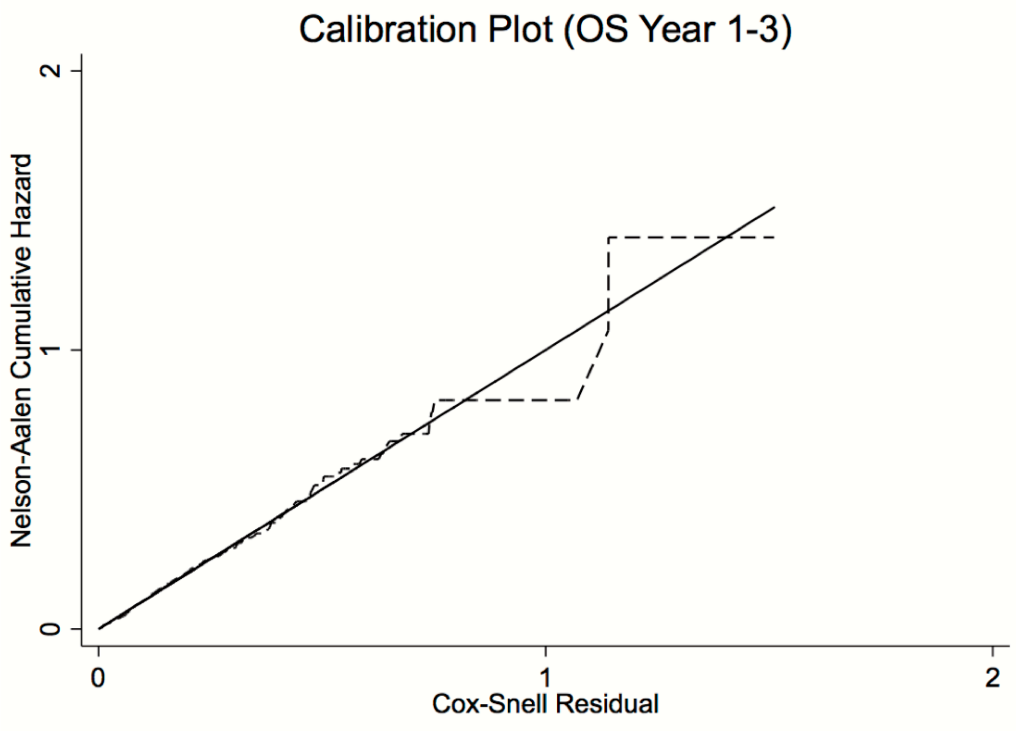

Supplemental Figure S5

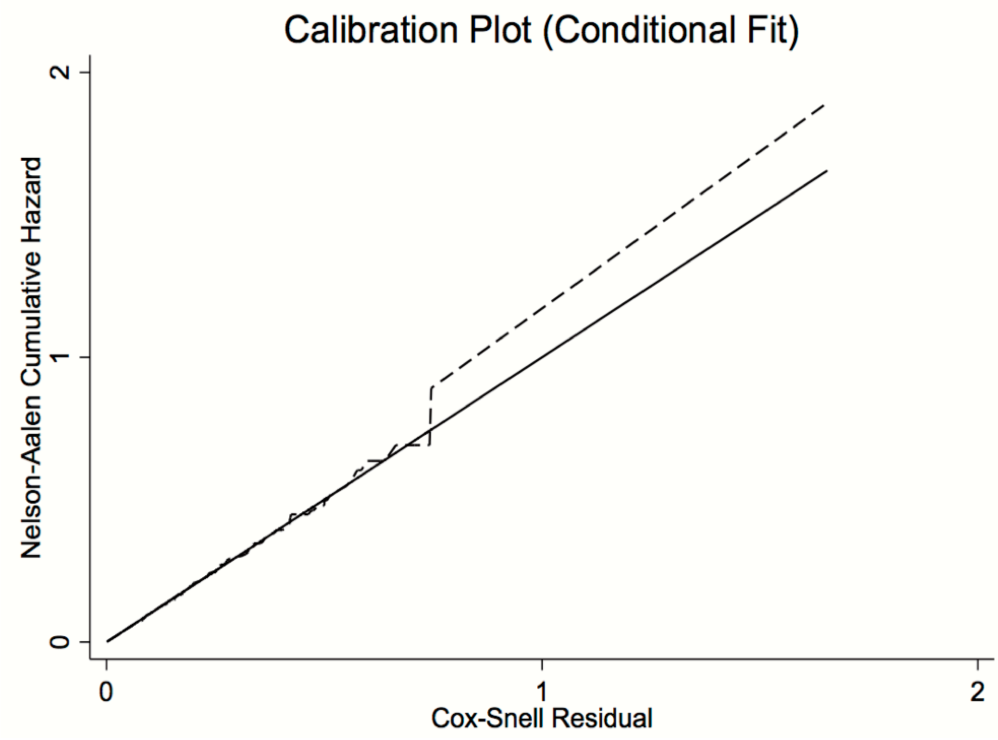

Supplement: Supplementary Information [file srep43928-s1.pdf]
